# 10-Year Retrospective Review of the Etiologies for Meningitis With Elevated Adenosine Deaminase in Cerebrospinal Fluid: Etiologies Other Than TB

**DOI:** 10.3389/fcimb.2022.858724

**Published:** 2022-07-05

**Authors:** Joomee Song, Si-Ho Kim, Yi-Rang Jung, Junsu Choe, Cheol-In Kang, Ju-Hong Min

**Affiliations:** ^1^ Department of Neurology, Samsung Medical Center, Sungkyunkwan University School of Medicine, Seoul, South Korea; ^2^ Neuroscience Center, Samsung Medical Center, Seoul, South Korea; ^3^ Division of Infectious Diseases, Samsung Changwon Hospital, Sungkyunkwan University School of Medicine, Changwon, South Korea; ^4^ Strategy Innovation Team, Samsung Medical Center, Seoul, South Korea; ^5^ Division of Pulmonary and Critical Care Medicine, Department of Medicine, Samsung Medical Center, Sungkyunkwan University School of Medicine, Seoul, South Korea; ^6^ Division of Infectious Diseases, Samsung Medical Center, Sungkyunkwan University School of Medicine, Seoul, South Korea; ^7^ Department of Health Sciences and Technology, Samsung Advanced Institute for Health Sciences and Technology (SAIHST), Sungkyunkwan University, Seoul, South Korea

**Keywords:** tuberculosis, meningitis, lymphoma, tuberculous meningitis, viral meningitis, adenosine deaminase (ADA)

## Abstract

**Purpose:**

An elevated adenosine deaminase (ADA) level in the cerebrospinal fluid (CSF) is considered a reliable marker of tuberculous meningitis (TBM). However, CSF-ADA levels can also be elevated in other diseases. We aimed to find the most common diagnosis of patients with elevated CSF-ADA levels for the last 10 years.

**Methods:**

We retrospectively investigated the diagnoses of all patients with elevated CSF-ADA (ADA ≥ 10 IU/L) levels between 2010 and 2019 at the Samsung Medical Center. Definite TBM was defined based on microbiological evidence. Clinical TBM was defined based on the brain imaging and response to the standard TB treatment. We compared the laboratory characteristics of the three most common diagnoses.

**Results:**

CSF-ADA levels were elevated in 137 (5.6%) of 2,600 patients. The most common diagnoses included hematologic malignancy (HM; n = 36, 26.2%), TBM (n = 26, 19.0%), and viral meningitis (VM; n = 25, 18.2%). CSF-ADA levels did not differ significantly between TBM [median (interquartile range (IQR)), 20.2 IU/L (13.8–29.3)] and HM [16.5 (12.8–24.0)]. However, CSF-ADA levels were lower in VM [14.0 (11.0–16.1)] than in TBM (p = 0.027). Lymphocyte-dominant pleocytosis was more common in VM [77.0% (70.8–81.5)] than in TBM [16.0 (3.0–51.0), p = 0.015] or HM [36.0 (10.0–72.0); p = 0.032]. Interestingly, the CSF characteristics of clinical TBM were similar to those of VM but not definite TBM.

**Conclusion:**

The most common diagnoses with elevated CSF-ADA levels were HM, followed by TBM and VM. Clinicians should carefully consider the differential diagnoses in patients with elevated CSF-ADA levels, especially those in the early stage of meningitis without microbiological evidence for TBM.

## Introduction

Tuberculous meningitis (TBM) is one of the most lethal and debilitating forms of TB that presents with few specific symptoms but needs an early diagnosis (Wilkinson et al., 2017). As a diagnostic clue, adenosine deaminase (ADA) could be increased in the cerebrospinal fluid (CSF) of patients with TBM due to activation of T-lymphocytes in response to tubercle bacillus infection. ADA is an enzyme found in most body cells and involved in purine catabolism and the cell-mediated immune (CMI) response related to T-lymphocyte activation (Ciruela et al., 1996; Gupta et al., 2010; Martinez-Navio et al., 2011). A meta-analysis reported that the pooled sensitivity and specificity of CSF-ADA for the diagnosis of TBM were 89% and 91%, respectively (the cutoff values ranged from 6 to 15 IU/L) (Pettersson et al., 1991; Pormohammad et al., 2017; Ekermans et al., 2017), which suggests it is a reliable and fast diagnostic tool for TBM given the poor sensitivity of acid-fast staining and molecular assays for the CSF (Ho et al., 2013; Mai and Thwaites, 2017).

Nevertheless, CSF-ADA levels are frequently elevated in various meningitis forms caused by other infectious or non-infectious etiologies including malignancies (López-Cortés et al., 1995, Ekermans et al., 2017; Hong et al., 2017). Consequently, it is challenging for clinicians to decide to start anti-TB treatment in undetermined meningitis with elevated CSF-ADA, especially when meningitis patients in the areas with a high prevalence of TB show elevated CSF-ADA levels without any disease-specific symptoms or laboratory results. Moreover, anti-TB medication puts a heavy strain on patients, since the treatment time is long and adverse reactions to it are relatively common (Yang et al., 2017). Therefore, it is necessary to know the detailed epidemiology of meningitis with elevated CSF-ADA as well as TBM and give TB medication to patients only when appropriate.

Therefore, it is necessary to know how common other etiologies of meningitis with elevated CSF-ADA rather than TBM. Herein, we evaluated the diversity of diagnoses and their contribution to the patients with meningitis with elevated CSF-ADA levels. Moreover, we compared the clinical and laboratory characteristics of meningitis with different diagnoses. This study will help clinicians diagnose and decide treatment plans for those patients.

## Methods

### Study Population and Period

Patients with meningitis who underwent testing for CSF-ADA were retrospectively reviewed between January 2010 and December 2019 at the Samsung Medical Center, which is a 1,950-bed, tertiary-care referral hospital located in Seoul, South Korea. Among them, patients with CSF-ADA levels ≥10 IU/L were included as patients with elevated CSF-ADA in this study, based on prior studies (Jasani et al., 2011; Pormohammad et al., 2017). The cutoff was set majorly based on the “Korean Guidelines for the management of TB, 4^th^ edition,” which reported the cutoff as 5–10 IU/L in Korea (Shim, 2020); the highest value, 10 IU/L, was selected to make the positive predictive value (PPV) and specificity as high as possible.

Regarding patients who were tested for CSF-ADA more than one time, the highest value among them was selected to include as many patients as possible. Patients with suspected intracranial hemorrhage [CSF–red blood cells (RBCs) ≥10,000/mm^3^] were excluded from etiology analysis because ADA could be falsely elevated due to the hemorrhage (Gorchynski et al., 2007).

### Definition, Data Collection, and Study Outcomes

The included patients’ diagnoses were reviewed with the medical records and classified according to criteria set based on the former guidelines and studies. In our medical institution, the tests for a patient with meningitis included the following: CSF cytology, Gram stain and culture, acid-fast stain and culture, fungus culture, PCR for TB, PCR for the virus (herpes simplex virus [HSV], varicella-zoster virus [VZV], Epstein–Barr virus [EBV], cytomegalovirus [CMV], and Japanese encephalitis virus [JEV]), PCR for protozoa (toxoplasma), and serologic test (serologic test for syphilis, IgM virus-specific antibody for JEV, and toxoplasma antibody).

The definite TBM were defined as meningitis with positive Acid Fast Bacilli culture for TB or positive nucleic acid amplification test (NAAT) for TB. The NAAT for TB was performed using the GENEDIA MTB/NTM Detection Kit (Green Cross Medical Science Corp., Chungbuk, Korea) or Xpert MTB/RIF (Cepheid, Sunnyvale, CA, USA). Clinical TBM was defined as follows: 1) meningitis with brain MRI findings suggestive of TBM (i.e., basal cistern enhancement with enhancing exudates) or evidence of extra-neural TB (e.g., miliary TB of the lung), 2) negative results in a biological test of all other etiologies, and 3) responded well to at least 6 months of empirical TB treatment (Botha et al., 2012; Solari et al., 2013). Definite viral meningitis (VM) was defined as meningitis with positive PCR or antibody tests for the viruses in the CSF, in the absence of other possible etiologies. Clinical VM was defined as follows: 1) meningitis with brain MRI findings suggestive of viral infection (e.g., temporal lobe enhancement in HSV infection) or CSF chemistry and cell count suggestive of viral infection (i.e., normal glucose level and mononuclear pleocytosis) (Cantu and Das, 2021), 2) negative results in the PCR or antibody tests for viruses, and 3) negative results in non-viral etiologies. Definite non-viral infectious meningitis was defined as meningitis with positive cultures, NAAT, or antibodies for the bacterial, fungal, toxoplasma, and non-tuberculous mycobacterial infections. Clinical non-viral infectious meningitis was defined as meningitis with brain MRI findings suggestive of meningitis (e.g., leptomeningeal enhancement in contrast to brain MRI not pathognomonic for TBM or VM), and CSF chemistry and cell count suggestive of non-viral infection (i.e., lower glucose level and neutrophil pleocytosis) (Cantu and Das, 2021) but negative test results in of viral or non-viral etiologies.

Definite meningeal involvement of hematologic malignancy (HM) or solid cancer was defined as meningitis with positive malignant cells in CSF or brain biopsy. Clinical meningitis due to malignancy was defined as meningitis in patients with hematologic malignancy or solid cancer without positive malignant cells in CSF or brain biopsy and without any evidence of all other meningitis etiology.

Moreover, the following data were collected from the study population’s electronic medical records: age, sex, and CSF analyses (ADA, RBCs, white blood cells [WBCs], pH, glucose, protein, and lactate dehydrogenase levels).

The primary aim of this study was to investigate the various possible diagnoses in patients with meningitis with elevated CSF-ADA levels. Subsequently, the sensitivity, specificity, negative predictive value (NPV), and PPV of CSF-ADA for TBM were calculated. The secondary aim of this study was to compare the clinical and laboratory characteristics of the three most common diagnoses: considering that the clinical diagnosis could be biased, only the definitive diagnoses with microbiological evidence were compared. Moreover, the CSF characteristics of definite TBM, clinical TBM, and definite VM were compared, since each of these diseases can present with lymphocyte-dominant pleocytosis in the CSF and can mimic the clinical manifestations of the others (Hong et al., 2017).

### Statistical Analysis

All statistical analyses were performed using a commercially available software package R 4.0.2 (Vienna, Austria; http://www.R-project.org). Student’s t-test or the Mann–Whitney U test was used to compare the continuous variables of the two groups, and the one-way ANOVA or Kruskal–Wallis test was used to compare the continuous variables of multiple groups. The chi-squared test or Fisher’s exact test was used to compare categorical variables. The Bonferroni method was used to conduct *post-hoc* analysis for multiple comparisons. All p-values were two-tailed, and p-values <0.05 were considered statistically significant.

## Results

### Diagnoses of Meningitis With Elevated Adenosine Deaminase Level in the Cerebrospinal Fluid

**Figure 1:**
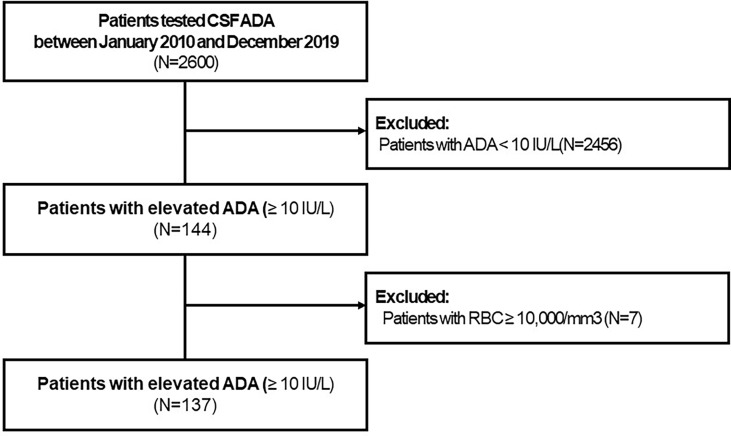
Flowchart to describe the enrolled study population. CSF, cerebrospinal fluid; ADA, adenosine deaminase; RBC, red blood cell.

Elevated CSF-ADA (≥10 IU/L) levels were observed only in 144 (5.6%) of the 2,600 eligible patients (**Figure 1**). Seven patients with CSF-RBCs ≥10,000/mm^3^ and elevated CSF-ADA were excluded. Among 137 eligible patients, the three most common diagnoses were HM (36/137, 26.3%), TBM (26/137, 19.0%), and VM (25/137, 18.2%) (**Table 1**). Definite HM, definite TBM, and definite VM were 32, 12, and 14, respectively. More specifically, lymphoma (28/32, 87.5%) was the most common etiology in the HM, while the JEV (6/14, 42.9%) and VZV (4/14, 28.6%) were the most common pathogens for VM.

**Table 1:** Definite and clinical diagnoses of patients with elevated ADA (≥10 IU/L).

Diagnosis	Patient number (%)	Remarks
**Tuberculous meningitis**	**26 (19.0)**	
Definite	12	6 cultures (+)/PCR (+), 5 cultures (+)/PCR (−) and 1 culture (−)/PCR (+)
Clinical	14	
**Viral meningitis**	**25 (18.2)**	
Definite	14	6 of JE virus, 4 of VZV, 2 of HSV, 1 of enterovirus and 1 of CMV meningitis with meningoencephalitis
Clinical	11	
**Non-viral infectious meningitis**	**22 (16.1)**	
Definite	14	10 bacterial, 1 fungal, 1 toxoplasma and 2 NTM
Clinical	8	
**CNS involvement of** **Hematologic malignancy**	**36 (26.2)**	
Definite	32	28 of lymphoma, 3 of leukemia, and 1 of multiple myeloma with CNS involvement
Clinical	4	
**CNS involvement of** **Solid cancer**	**4 (2.9)**	
Definite	2	1 of breast cancer, 1 of lung cancer
Clinical	2	
**Others**	**2 (1.5)**	
**Unknown origin**	**19 (13.9)**	
**Multiple diagnoses**	**3 (2.2)**	1 of *Klebsiella* meningitis with HSV, 1 of enterococcal meningitis with IgG4-related disease, and 1 of fungal meningitis with lymphoma CNS involvement
**Total**	**137 (100)**	

JE, Japanese encephalitis; VZV, varicella-zoster virus; HSV, herpes simplex virus; CMV, cytomegalovirus; NTM, non-tuberculous mycobacteria; CNS, central nervous system; ADA, adenosine deaminase. Bold values, Data are presented as n (%) or n.

The proportion of clinical and definitive TBM was significantly higher (p < 0.001) in the patients with elevated CSF-ADA (26/144, 18.1%) than in the patients without CSF-ADA (14/2,456, 0.6%). The calculated PPV of CSF-ADA for TBM was only 19.2%. The sensitivity, specificity, and NPV of CSF-ADA for TBM diagnosis were calculated as 65%, 95.4%, and 99.4%, respectively. We have specified the sensitivity, specificity, PPV, and NPV of CSF-ADA for the three most common diagnoses (i.e., TBM, HM, and VM) in **Supplementary Table 2**.

### Comparison of Adenosine Deaminase Level in the Cerebrospinal Fluid and Other Characteristics Among Hematologic Malignancy, Tuberculous Meningitis, and Viral Meningitis

**Figure 2:**
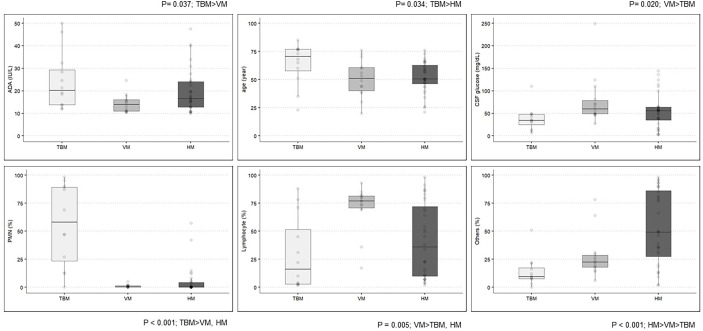
Clinical and laboratory characteristics among the definite TBM, VM, and HM groups. ADA, adenosine deaminase; TBM, tuberculous meningitis; VM, viral meningitis; HM, hematologic malignancy; CSF, cerebrospinal fluid; PMN, polymorphonuclear neutrophils.

No significant difference was observed in the level of CSF-ADA between TBM [median (interquartile range (IQR)), 20.20 IU/L (13.78–29.30)] and HM [16.05 (12.80–24.02)], while the CSF-ADA levels were significantly lower in VM [14.00 (10.95–16.07)] than in TBM (p = 0.027). Regarding the cell count of the CSF, the prevalence of lymphocyte-dominant pleocytosis was higher in VM [77.0% (70.8–81.5)] than in TBM [16.0% (3.0–51.0), p = 0.015] or HM [36.0% (10.0–72.0); p = 0.032], while the prevalence of polymorphonuclear neutrophil-dominant pleocytosis was higher in TBM [58.0% (23.5–89.3)] than in VM [1.0% (0.0–1.0)] or HM [0.0% (0.0–4.3)]. As expected, CSF glucose levels were lower in TBM [34.5 (25.3–48.0)] than in VM [59.5 (49.0–78.8), p = 0.002] (**Figure 2** and **Supplementary Table 1**). Demographics-wise, patients in the TBM group were the oldest (median age, 70.5, 51.0, and 50.5 years in the TBM, VM and HM groups, respectively; p = 0.034).

### Comparison of Cerebrospinal Fluid Characteristics Among the Definite Tuberculous Meningitis, Clinical Tuberculous Meningitis, and Definite Viral Meningitis Groups

CSF-ADA levels were higher in definite TBM [20.20 IU/L (13.78–29.30)] than in clinical TBM [13.65 (11.57–17.48), p = 0.024]. Moreover, CSF glucose was lower in definite TBM [59 mg/dl (49.25–76)] than in clinical TBM [34.5 (25.25–48), p = 0.008]. In general, the CSF characteristics of clinical TBM were similar to those of definite VM, rather than definite TBM (**Table 2**). Lymphocyte-dominant pleocytosis was more dominant in clinical TBM than in definite TBM (p < 0.001).

**Table 2:** Patient characteristics and cerebrospinal fluid analysis from the definite diagnosis of viral meningitis and clinical diagnosis of tuberculous meningitis.

	Clinical TBM (14)	Definite TBM (12)	P-value	Definite VM (14)	P-value
Patient characteristics					
Age	43.5 (35.5–54.0)	70.5 (57.75–77.0)	0.039	51.5 (40.25–60.75)	0.514
Sex (male), N (%)	12 (85.7)	3 (25.0)	0.004	6 (42.9)	0.046
CSF analysis					
ADA (IU/L)	13.65 (11.57–17.48)	20.20 (13.78–29.30)	0.024	14.00 (10.95–16.07)	0.713
RBC (/mm^3^)	7 (2.5–14.25)	30 (11.25–94)	0.054	11 (2.25–69.5)	0.613
WBC (/mm^3^)	95 (76.25–126.25)	170.0 (72.5–360)	0.425	130.0 (52.25–340)	0.800
PMN (%)	0.5 (0–2.75)	58 (23.5–89.25)	0.001	1 (0–1)	0.751
Lymphocyte (%)	87 (76.50–89.75)	16 (3–51.5)	0.001	77 (70.75–81.5)	0.085
Eosinophil (%)	0 (0–0)	0 (0–0)	0.105	0 (0–0)	0.290
Other types (%)	11 (9.25–13)	9.5 (7.75–17.25)	0.718	22.5 (18–28.5)	<0.001
pH	7.20 (7.20–7.30)	7.20 (7.18–7.30)	0.441	7.25 (7.20–7.30)	0.528
Glucose (mg/dl)	59 (49.25–76)	34.5 (25.25–48)	0.008	59.5 (49–78.75)	0.890
Protein (mg/dl)	113.60 (72.12–131.35)	181.95 (83.58–292.93)	0.160	122.95 (89.45–157.43)	0.603
LDH (U/L)^a^	109.0 (65.0–141.5)	166.0 (108.0–524.5)	0.139	162.5 (116.2–220.0)	0.256

Data are presented as the median value (interquartile range), unless otherwise specified.

TBM, tuberculous meningitis; VM, viral meningitis; CSF, cerebrospinal fluid; ADA, adenosine deaminase; RBC, red blood cell; WBC, white blood cell; PMN, polymorphonuclear neutrophil; LDH, lactate dehydrogenase.

a The LDH data were available for only 11, 10, and 6 patients with clinical TBM, definite TBM, and definite VM, respectively.

## Discussion

Our study found that HM, TBM, and VM were the three most common diagnoses in patients with elevated CSF-ADA levels for the past 10 years in our institution, which is the major tertiary hospital in Korea. More than two-thirds of patients with meningitis with elevated CSF-ADA levels did not have TBM. Moreover, the CSF findings of clinical TBM were similar to those of definite VM and not definite TBM. Therefore, this study suggests that clinicians should consider various diagnoses in patients with elevated CSF-ADA levels without any determined etiology.

In our study, meningeal involvement in HM, especially lymphoma, was the most common cause of CSF-ADA elevation. As mentioned above, ADA is a marker of lymphocytic activation associated with the CMI (Ciruela et al., 1996; Martinez-Navio et al., 2011). Therefore, CSF-ADA levels could be elevated in the event of CMI activation caused by lymphoproliferative malignancy. Although most recent studies have focused on distinguishing between TBM and meningitis of other bacterial or infectious origins, an early 1990s study already reported that the concentration of CSF-ADA could be elevated in patients with lymphoma (5 patients, range, 4–25 IU/L) like in TBM (3 patients, range, 20–23 IU/L) (Pettersson et al., 1991). Lymphoma and TBM are representative diseases whose clinical manifestations can mimic each others'’, or the two can exist concomitantly (Falagas et al., 2010; Tai et al., 2020). In these circumstances, CSF-ADA is not a very useful diagnostic parameter, and clinicians usually experience more trouble in distinguishing between TBM and HM, than between TBM and bacterial meningitis.

VM was the third most common cause of meningitis in patients with elevated CSF-ADA levels in our study. The VM's level of CSF-ADA overlapped with the TBM's. Moreover, the CSF findings of clinical TBM were similar to those of definite VM, and not definite TBM. This finding is not unusual because cell-mediated immunity plays a pivotal role in the defense against viral infections in humans (Zajac and Harrington, 2008). Also, this evinces the possibility of the existence of a significant difference between the actual diagnosis and the clinician’s diagnosis based on the empirical belief that meningitis with lymphocyte-dominant pleocytosis and elevated CSF-ADA level is TBM. These results are consistent with a series of prior studies that have reported elevated ADA levels in patients with VM (Cho et al., 2013; Ekermans et al., 2017; Hong et al., 2017). Hong et al. reported a study that compared CSF-ADA levels of VZV meningitis, VZV meningitis previously misdiagnosed as true TBM. In that study, the ADA level of the misdiagnosed groups (range, 8.0–15.0 IU/L) overlapped with that of the true TBM group (range, 11.7–31.6 IU/L), while the proportion of lymphocytosis (median, 83% versus 60% in the misdiagnosis versus the true TBM group, respectively; p < 0.001) was the significant difference between the misdiagnosed and true TBM groups (Hong et al., 2017). Clinicians should be aware that misdiagnosis and inappropriate treatment of meningitis can occur if they depend heavily on the ADA findings when patients are without etiology-specific symptoms or signs.

Meanwhile, our study showed that Japanese encephalitis was the most common etiology of definite VM with elevated CSF-ADA levels. This finding is interesting since CSF-ADA elevation has not been studied in patients with Japanese encephalitis. We suggest considering Japanese encephalitis as a differential diagnosis in patients with elevated CSF-ADA levels with meningitis with an undiscovered etiology.

In this study, the PPV of CSF-ADA for TBM was 18.1% (cutoff value, 10 IU/L). The PPV was even lower for definite TBM, at only 8.3%. The low PPV of CSF-ADA in this study is a salient finding considering PPV depends on the prevalence of a given disease in the study population: TB is endemic to the Republic of Korea (70/100,000 of the population), which has a 23-fold higher incidence of TB as compared to the United States (3.1/100,000 population) (Heeae et al., 2019). Therefore, prior 1:N matched studies that compared TBM with bacterial or VM without considering prevalence may have distorted the real-world incidence. Even if the sensitivity and specificity of CSF-ADA were 89% and 91%, respectively, as demonstrated by a previous meta-analysis, the PPV is only 8.2% when the prevalence of TBM is supposed to be 1%. Considering the low prevalence of TBM worldwide (1% among patients with pulmonary TB) (Wilkinson et al., 2017), the proportion of TBM in the meningitis population with elevated CSF-ADA levels might be very low, especially in a non-TB endemic area.

Seventy percent of the TBM has been diagnosed between 2010 and 2014, while 70% of the HM and VM has been diagnosed between 2015 and 2019. This reflects the constant decrement in TB prevalence (per 100,000) during the past 10 years in Korea; the prevalence was 152 in 2010, 101 in 2015, and 59 in 2019 (Prevention, K. C. F. D. C. A, 2020). In contrast, the incidence (per 100,000) of malignancy in South Korea has increased from 417.6 in 2010 to 496.2 in 2019 (Korea, S, 2020; Center N.C., 2020). Therefore, the weight of the ADA elevation in CSF should be lowered in diagnosing TBM these days.

Our study has some limitations. First, it was a single-center, retrospective study: the differential diagnosis could have been different depending on the type of hospital and geographical characteristics. However, our medical center is the third largest tertiary referral center in South Korea for all serious illnesses, making it a suitable representation of the whole nation. Therefore, low prevalence or pretest probability of TBM is a major factor to decrease the diagnostic performance of ADA in general clinical situations, not limited to our medical institution. Second, clinical diagnoses may possibly differ from the actual underlying etiology. Twenty-eight percent (39/137) of patients were provided only with clinical diagnoses, without specific microbiological evidence on CSF analysis. Therefore, the true prevalence of each etiology should be cautiously interpreted. Finally, the similarity between the CSF findings of TBM and definite VM does not represent direct evidence of the possibility of misdiagnosing VM as TBM. Further studies are needed to examine the definitive microbiological evidence of patients diagnosed with clinical TBM. In addition, definitive TBM cases in our study showed relatively neutrophil-dominant pleocytosis. Although lymphocyte-dominant pleocytosis is known for characteristic findings in patients with TB meningitis, neutrophil-dominant pleocytosis can be frequently observed in TB meningitis, especially in the early stage of disease progression (Sþtlaş et al., 2003). Several studies, which evaluated the clinical diagnostic rules for the TBM, suggested that one of the predictors of TBM was CSF leukocytosis with a proportion of neutrophils lower than 50%–90% (Wilkinson et al., 2017). This range of neutrophilic proportions is consistent with our study findings.

In conclusion, patients with meningitis and elevated CSF-ADA levels could have a variety of diagnoses: elevated CSF-ADA levels in these patients were not specific to TBM, as evidenced by the low PPV. Moreover, patients with clinical TBM who improved with standard TB treatment showed CSF findings that were similar to those with definite VM, but not akin to those with definite TBM. Hence, non-TBM patients may undergo unnecessary TB treatment if clinicians depend on CSF-ADA to diagnose TBM. Clinicians should consider the overall clinical and laboratory findings and sufficient alternative diagnoses in meningitis with elevated CSF-ADA levels.

## Data Availability Statement

All data generated or analysed during this study are included in this published article (and its supplementary information files).

## Ethics Statement

This study was approved by the Institutional Review Board of the Samsung Medical Center (IRB number: SMC 2021-01-122). Written informed consent for participation was not required for this study in accordance with the national legislation and the institutional requirements.

## Author Contributions

JS and S-HK equally conceived the study; acquired, analyzed, and interpreted the data; and drafted and revised the manuscript. J-HM conceived, designed, and supervised the study; interpreted the data; and drafted and revised the manuscript for intellectual content. Y-rJ interpreted and analyzed the data. JC revised the manuscript for intellectual content. C-IK revised the manuscript for intellectual content and supervised the study. All authors listed have made a substantial, direct, and intellectual contribution to the work and approved it for publication.

## Conflict of Interest

The authors declare that the research was conducted in the absence of any commercial or financial relationships that could be construed as a potential conflict of interest.

## Publisher’s Note

All claims expressed in this article are solely those of the authors and do not necessarily represent those of their affiliated organizations, or those of the publisher, the editors and the reviewers. Any product that may be evaluated in this article, or claim that may be made by its manufacturer, is not guaranteed or endorsed by the publisher.

## Supplementary Material

Supplementary Table 1 Patient characteristics and cerebrospinal fluid analysis in definite tuberculous meningitis, viral meningitis, and hematologic malignancy with CNS involvement. ^a^ The LDH data were available for only 10, 6, and 28 patients with definite TBM, VM, and HM, respectively. Data represent median value (interquartile range), unless otherwise specified. HM, hematologic malignancy; CSF, cerebrospinal fluid; ADA, adenosine deaminase; RBC, red blood cell; WBC, white blood cell; PMN, polymorphonuclear neutrophil; LDH, lactate dehydrogenase, CNS, central nervous system.

Supplementary Table 2 Sensitivity, specificity, PPV and NPV of CSF-ADA for three most common diagnoses (i.e., TBM, HM, VM)TBM, tuberculous meningitis, HM, hematologic malignancy, VM, Viral meningitis.
