# Supplementary material for: 10-Year Retrospective Review of the Etiologies for Meningitis With Elevated Adenosine Deaminase in Cerebrospinal Fluid: Etiologies Other Than TB
Source: Front Cell Infect Microbiol. 2022 Jul 5;12:858724. doi: 10.3389/fcimb.2022.858724 (PMC9295904; doi:10.3389/fcimb.2022.858724)
Supplement: Supplementary Table 1 — Patient characteristics and cerebrospinal fluid analysis in definite tuberculous meningitis, viral meningitis, and hematologic malignancy with CNS involvement. a The LDH data were available for only 10, 6, and 28 patients with definite TBM, VM, and HM, respectively. Data represent median value (interquartile range), unless otherwise specified. HM, hematologic malignancy; CSF, cerebrospinal fluid; ADA, adenosine deaminase; RBC, red blood cell; WBC, white blood cell; PMN, polymorphonuclear neutrophil; LDH, lactate dehydrogenase, CNS, central nervous system. [file DataSheet_1.docx]

**Supplementary Table 1. Patient characteristics and cerebrospinal fluid analysis in definite tuberculous meningitis, viral meningitis, and hematologic malignancy with CNS involvement**

|  | Tuberculosis  (12) | Virus  (14) | Hematologic malignancy  (32) | p value | Post-hoc analysis |
| --- | --- | --- | --- | --- | --- |
| Patient characteristics |  |  |  |  |  |
| Age | 70.5 (57.75-77) | 51.0 (40.25-60.75) | 50.5 (46.25-62.75) | 0.034 | Tuberculosis>HM |
| Sex (male), N (%) | 3 (25.0) | 6 (42.9) | 18 (56.3) | 0.170 |  |
| CSF analysis |  |  |  |  |  |
| ADA (IU/L) | 20.20 (13.78-29.30) | 14.00 (10.95-16.07) | 16.50 (12.80-24.02) | 0.037 | Tuberculosis>Virus |
| RBC (/mm^3^) | 30 (11.25-94) | 11 (2.25-69.5) | 3 (1 – 117.75) | 0.265 |  |
| WBC (/mm^3^) | 170 (72.5-360) | 130 (52.25-340) | 53.5 (11.5-201.25) | 0.276 |  |
| PMN (%) | 58 (23.5-89.25) | 1 (0-1) | 0 (0-4.25) | <0.001 | Tuberculosis>Virus, HM |
| Lymphocyte (%) | 16 (3-51.5) | 77 (70.75-81.5) | 36 (10-72) | 0.005 | Virus>Tuberculosis, HM |
| Eosinophil (%) | 0 (0-0) | 0 (0-0) | 0 (0-0) | 0.208 |  |
| Other types (%) | 9.5 (7.75-17.25) | 22.5 (18-28.5) | 49 (27.5-86) | <0.001 | HM>Virus>Tuberculosis |
| pH | 7.20 (7.18-7.30) | 7.25 (7.20-7.30) | 7.30 (7.20-7.30) | 0.299 |  |
| Glucose (mg/dL) | 34.5 (25.25-48) | 59.5 (49-78.75) | 55.5 (35.25-64) | 0.020 | Virus>Tuberculosis |
| Protein (mg/dL) | 181.95 (83.58-292.93) | 122.95 (89.45-157.43) | 155.75 (78.97-415.25) | 0.504 |  |
| LD (U/L) ^a^ | 166.0 (108.0-524.5)  NA 2 | 162.5 (166.2-220.0)  NA 8 | 250.0 (130.8-433.2)  NA 4 | 0.681 |  |

^a^ The LD data were available for only 10, 6, and 28 patients with definite TBM, VM, and HM, respectively.

Data represent median value (interquartile range), unless otherwise specified.

HM, hematologic malignancy; CSF, cerebrospinal fluid; ADA, adenosine deaminase; RBC, red blood cell; WBC, white blood cell; PMN, polymorphonuclear neutrophil; LD, lactate dehydrogenase, CNS, central nervous system

**Supplementary Table 2. Sensitivity, specificity, PPV and NPV of CSF-ADA for three most common diagnoses (i.e., TBM, HM, VM)**

|  | **TBM** | **HM** | **VM** |
| --- | --- | --- | --- |
| Sensitivity | 0.65 | 0.073 | 0.071 |
| Specificity | 0.954 | 0.949 | 0.948 |
| PPV | 0.181 | 0.25 | 0.174 |
| NPV | 0.994 | 0.813 | 0.867 |

TBM, tuberculous meningitis, HM, hematologic malignancy, VM, Viral meningitis
